# Supplementary material for: Activation of CD8 T cells accelerates anti-PD-1 antibody-induced psoriasis-like dermatitis through IL-6
Source: Commun Biol. 2020 Oct 15;3:571. doi: 10.1038/s42003-020-01308-2 (PMC7567105; doi:10.1038/s42003-020-01308-2)
Supplement: Supplementary file 1 — Supplementary Information [file 42003_2020_1308_MOESM1_ESM.pdf]

Supplemental Figures

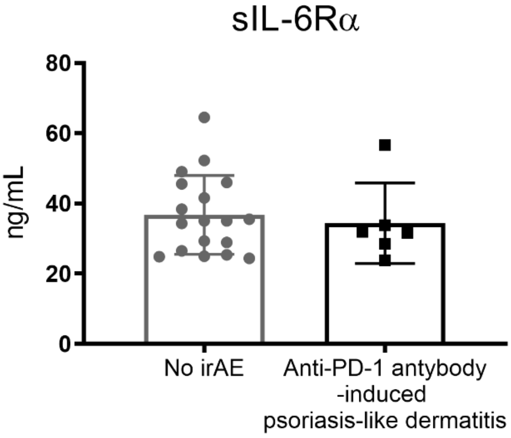

Supplemental Figure 1. Profiles of serum soluble interleukin-6 receptor alfa (sIL-6Rα) levels and the correlation between serum soluble IL-6 (sIL-6) and sIL-6Rα levels with human samples

Serum sIL-6Rα levels from anti-programmed cell death (PD)-1 antibody-treated cancer patients who developed psoriasis-like dermatitis as an immune-related adverse event (irAE, n = 6) and those with no irAE (n = 18). Note that there were two samples in psoriasis-like dermatitis group and one sample missing from serum sIL-6 analysis due to the shortage of samples remained.

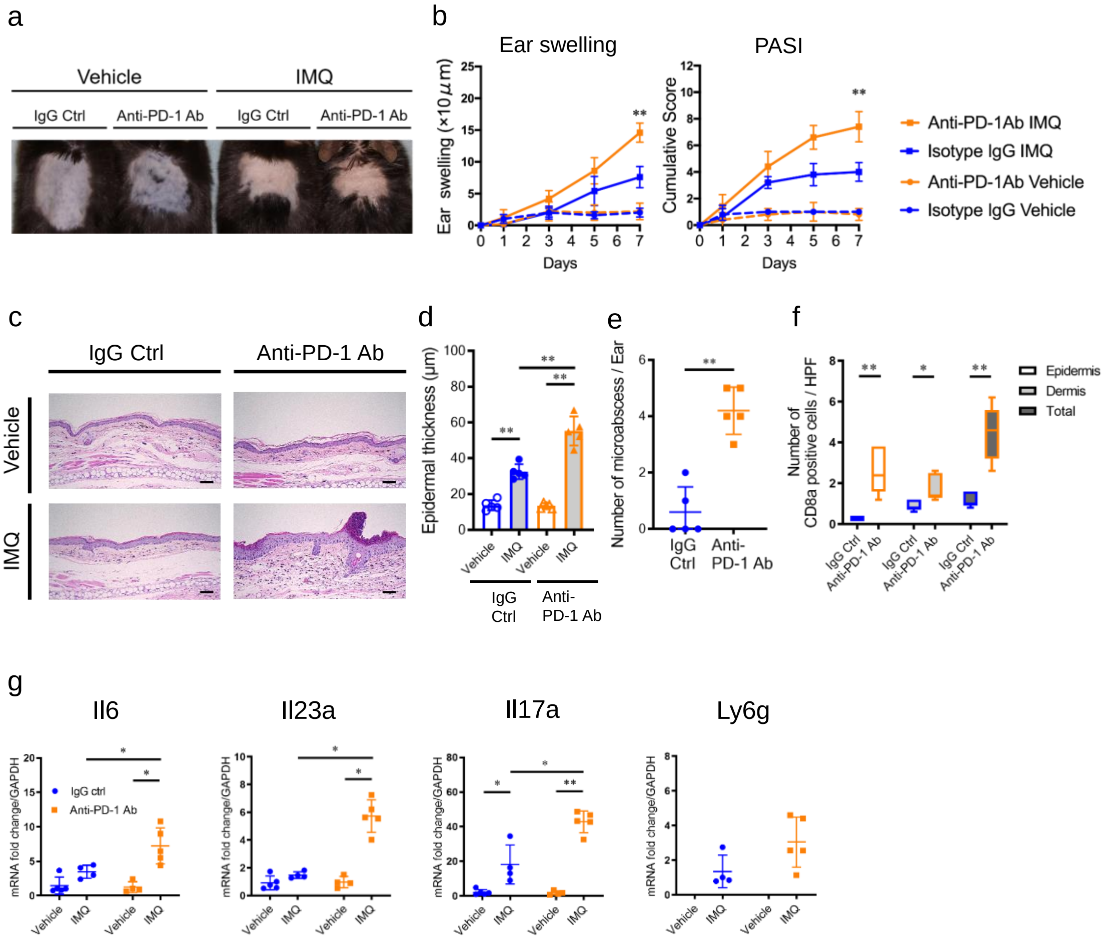

Supplemental Figure 2. Clinical and histological appearance and cytokine mRNA expression in imiquimod (IMQ)-induced psoriasis-like dermatitis in anti-PD-1 blocking antibody (Ab)-treated mice.

(A) Representative clinical images at day 7 of IMQ-induced psoriasis-like dermatitis in anti-PD-1 Ab (clone RMP1-14 [Bio X Cell, Lebanon, NH])-treated wild-type (WT) mice and rat IgG2a isotype control Ab (IgG Ctrl, Bio X Cell)-treated WT mice. The mice

received intraperitoneal injections with 200 μg of these antibodies on alternate days before the application of IMQ in accordance with previous study .

Application of vehicle cream was used as a control. (B) The course of ear swelling and PASI score representing the severity of erythema, scaling, and skin thickness. $^{**}P < 0.01$ by two-way ANOVA. (C) Representative images of hematoxylin-eosin (HE)-stained ear samples from IMQ-induced psoriasis-like dermatitis at day 7. Scale bars, 100 μm. (D, E) Epidermal hyperplasia (D), and the number of epidermal neutrophilic micro-abscesses (E) in the ear samples (n = 5 in each group). Data are shown as mean ± standard deviation (SD). $^{**}P < 0.01$ by nonparametric 2-tailed Mann-Whitney *U* test. (F) Immunohistochemistry analysis for the number of infiltrated CD8 T cells in the epidermis (n = 5 in each group). (G) Quantitative reverse transcriptase-polymerase chain reaction (qRT-PCR) analysis of psoriasis-related cytokines and the neutrophilic surface marker *Ly6g* in ear samples at day 7 (n = 4 - 5 in each group). Fold changes in mRNA levels were calculated and normalized against *GAPDH* mRNA levels. Data are expressed as mean ± SD. Data are representative of two independent experiments. $^{*}P < 0.05$, $^{**}P < 0.01$ by nonparametric 2-tailed Mann-Whitney *U* test.

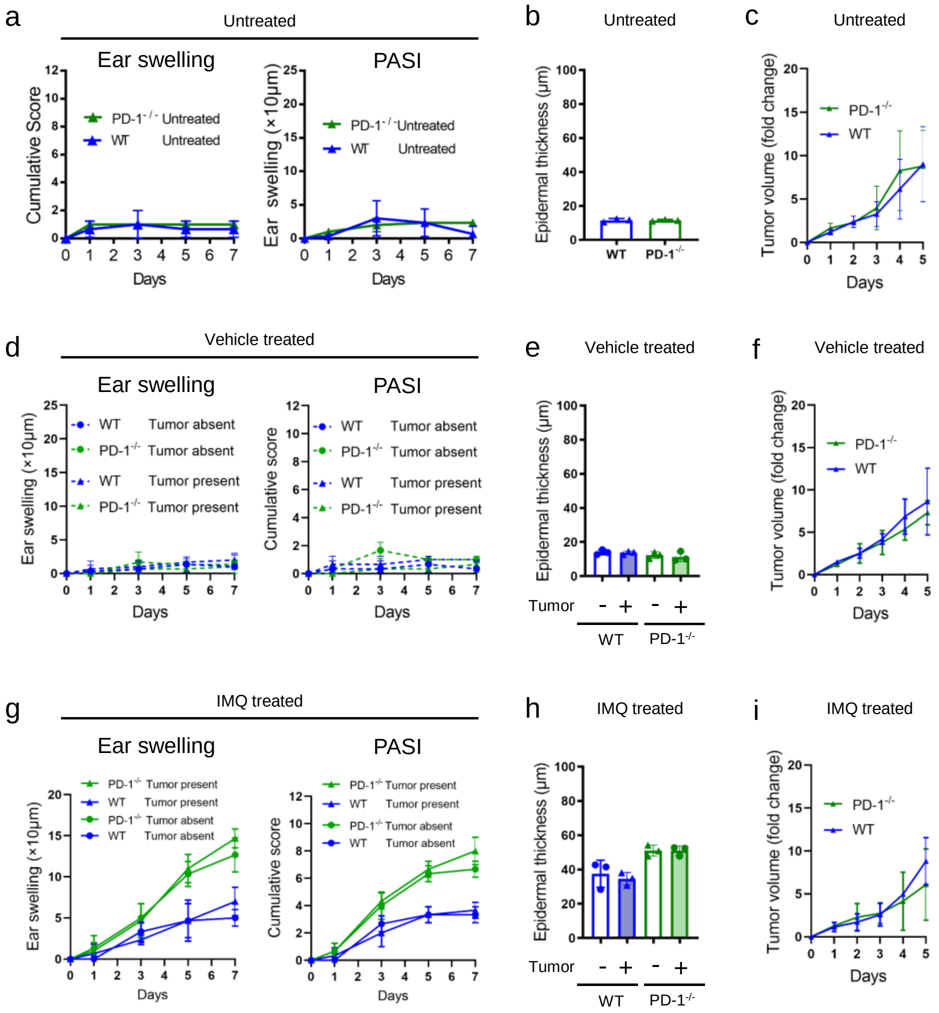

Supplemental Figure 3. Comparison of clinical and histological appearance of IMQ-induced psoriasis-like dermatitis with B16 melanoma mouse model

The course of ear swelling and PASI score representing the severity of erythema, scaling, and skin thickness (a, d, g), epidermal hyperplasia (b, e, h) and tumor growth kinetics of tumor (C, f, i) were evaluated in both WT and PD-$1^{-/-}$ mice, which were inoculated on their backs with 5 × 10^5 B16F10 murine melanoma cells (TKG 0348 from

Cell Resource Center for Biomedical Research, Tohoku University [Miyagi, Japan]), and classified into three groups on day 8 (untreated group, vehicle treated group, and IMQ treated group, n =3 each). Equal number of mice without tumor were also classified into three groups as controls. Each data are means ± SEM of three mice per group.

a

b

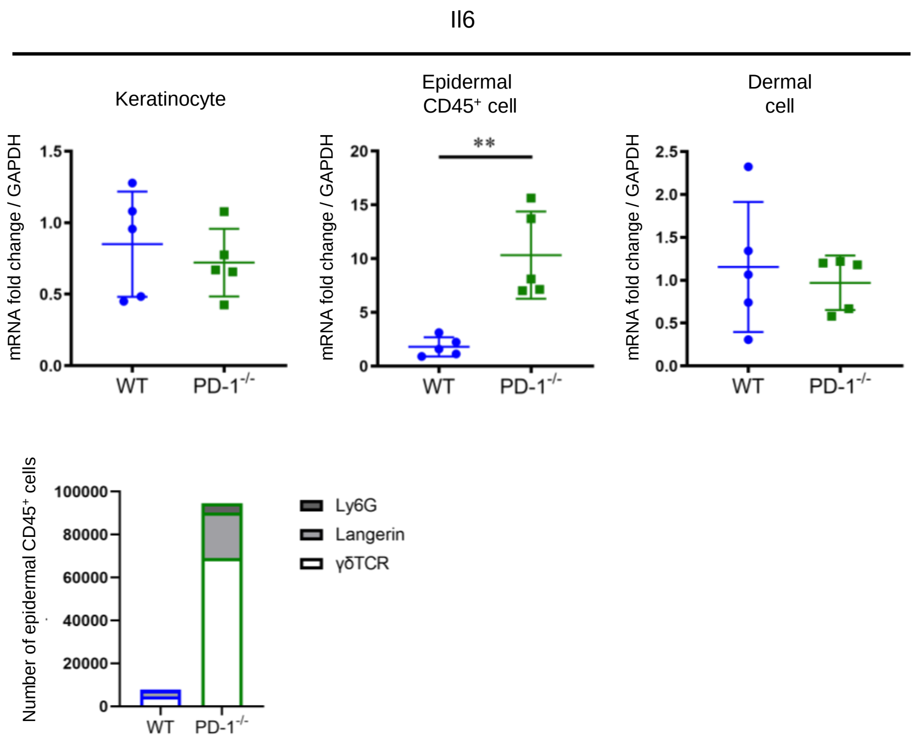

Supplemental Figure 4. PD-1 deficient mouse display enhanced IL-6 production on an epidermal CD45 positive cell population in IMQ-induced psoriasis-like dermatitis

(A) The qRT-PCR analysis of IL-6 on ear skin samples from IMQ-treated five mice per group, which were processed into keratinocyte, epidermal $CD45^+$ cell and dermal cell populations. $^{**}P < 0.01$ by nonparametric 2-tailed Mann-Whitney U test. (B) Cell counting and flow cytometry analysis revealed total numbers and distributions of IL-6 producible epidermal $CD45^+$ cell populations. Ly6G$^+$, Langerin$^+$, and γδ T-cell receptor (TCR)$^+$ cells represent neutrophils, Langerhans cells, and γδ T cells, respectively. Data represent the accumulation of five samples per group.

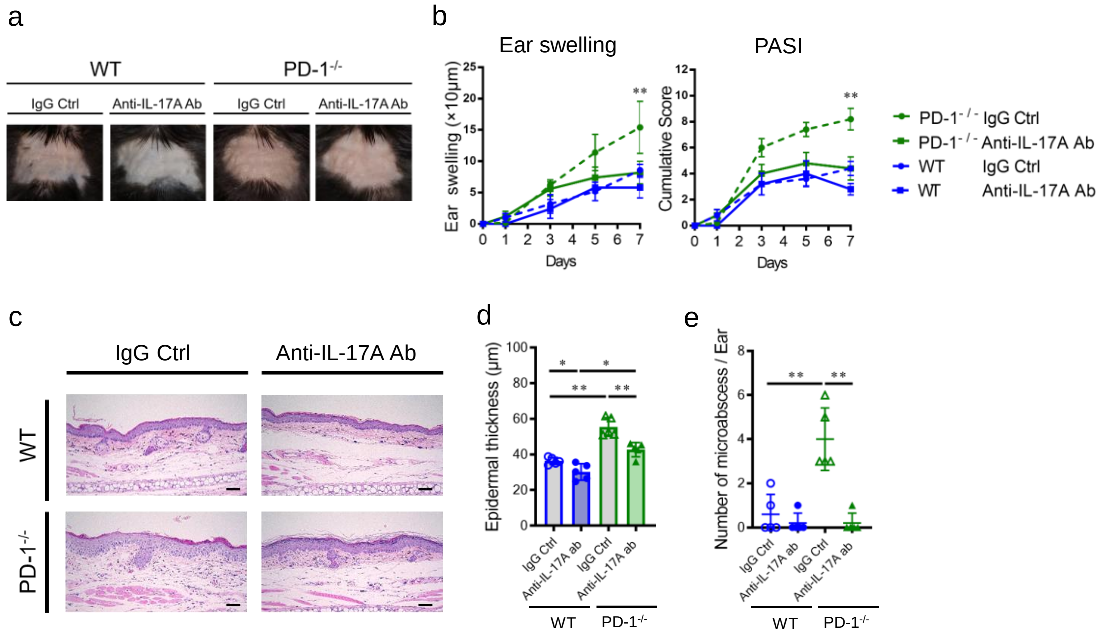

Supplemental Figure 5. Characteristics of neutralizing IL-17A monoclonal antibody-treated IMQ-induced psoriasis-like dermatitis in PD-1 deficient mice

(A) Representative clinical images of IMQ-induced psoriasis-like dermatitis at day 7 in both PD-1$^{-/-}$ mice and WT mice treated with neutralizing IL-17A Ab (clone 17F3, Bio X Cell) or murine IgG1 isotype Ctrl Ab (Bio X Cell). The mice were injected intraperitoneally with 200 μg of these Abs at the dose at days 0, 3, and 6 in accordance with previous study [46]. (B) Ear swelling and PASI score (n = 5 in each group). $^{**}P < 0.01$ by two-way ANOVA. (C) Representative HE staining of ear skin samples at day 7. Scale bars, 50 μm. (D) Epidermal thickness. (E) The number of epidermal, neutrophilic micro-abscess. Data are expressed as mean ± SEM. $^{*}P < 0.05$ and $^{**}P < 0.01$ by nonparametric 2-tailed Mann-Whitney *U* test.

Table 1 List of primers used in the present study

Primer	AssayID	Forward (5' - 3')	Reverse (5' - 3')
**Genotyping**			
Cre		GAACCTGATGGACATGTTCAGG	AGTGCGTTCGAACGCTAGAGCCTGT
PD-1 $^{-/-}$		TCTATGGAAGAATTTTGAGGGGAGATGG	GGGTGACATGTAAATGTGGAGAGAGCAT
PD-1 ^{loxP}		CAAGGACGACACTCTGGTGA	AGGCTCCCCATTGACTTCT
Pdcd1		CAC TAT CCC ACT GAC CCT TCA	CAC AGG GTA GGC ATG TAG CA
**qRT-PCR**			
Il6	Mm.PT.58.10005566		
Il23a	Mm.PT.58.10594618.g		
Il17a	Mm.PT.58.6531092		
Ifng	Mm.PT.58.41769240		
CD8a	Mm.PT.58.29971442		
Ly6g	Mm.PT.58.30498043		
CXCL9	Mm.PT.58.5726745		
