# Supplementary material for: Activation of CD8 T cells accelerates anti-PD-1 antibody-induced psoriasis-like dermatitis through IL-6
Source: Commun Biol. 2020 Oct 15;3:571. doi: 10.1038/s42003-020-01308-2 (PMC7567105; doi:10.1038/s42003-020-01308-2)
Supplement: Supplementary file 5 — Reporting Summary [file 42003_2020_1308_MOESM5_ESM.pdf]

nature research

Corresponding author(s): Naoko Okiyama

Last updated by author(s): Aug 27, 2020

Reporting Summary

Nature Research wishes to improve the reproducibility of the work that we publish. This form provides structure for consistency and transparency in reporting. For further information on Nature Research policies, see our Editorial Policies and the Editorial Policy Checklist.

Statistics

For all statistical analyses, confirm that the following items are present in the figure legend, table legend, main text, or Methods section.

n/a	Confirmed	
☐	☒	The exact sample size (n) for each experimental group/condition, given as a discrete number and unit of measurement
☐	☒	A statement on whether measurements were taken from distinct samples or whether the same sample was measured repeatedly
☐	☒	The statistical test(s) used AND whether they are one- or two-sided<br>*Only common tests should be described solely by name; describe more complex techniques in the Methods section.*
☐	☒	A description of all covariates tested
☐	☒	A description of any assumptions or corrections, such as tests of normality and adjustment for multiple comparisons
☐	☒	A full description of the statistical parameters including central tendency (e.g. means) or other basic estimates (e.g. regression coefficient) AND variation (e.g. standard deviation) or associated estimates of uncertainty (e.g. confidence intervals)
☐	☒	For null hypothesis testing, the test statistic (e.g. F, t, r) with confidence intervals, effect sizes, degrees of freedom and P value noted<br>*Give P values as exact values whenever suitable.*
☒	☐	For Bayesian analysis, information on the choice of priors and Markov chain Monte Carlo settings
☒	☐	For hierarchical and complex designs, identification of the appropriate level for tests and full reporting of outcomes
☒	☐	Estimates of effect sizes (e.g. Cohen's d, Pearson's r), indicating how they were calculated

Our web collection on statistics for biologists contains articles on many of the points above.

Software and code

Policy information about availability of computer code

Data collection	No software was used.
Data analysis	No software was used.

For manuscripts utilizing custom algorithms or software that are central to the research but not yet described in published literature, software must be made available to editors and reviewers. We strongly encourage code deposition in a community repository (e.g. GitHub). See the Nature Research guidelines for submitting code & software for further information.

Data

Policy information about availability of data

All manuscripts must include a data availability statement. This statement should provide the following information, where applicable:

- Accession codes, unique identifiers, or web links for publicly available datasets
- A list of figures that have associated raw data
- A description of any restrictions on data availability

Raw data for graphs can be found in Supplementary Data 1. CRISPR sgRNA sequences are available in Supplementary Data 2. All other data are available within the manuscript files or from the corresponding author upon reasonable request.

Field-specific reporting

Life sciences study design

All studies must disclose on these points even when the disclosure is negative.

Sample size	For human samples, the sample sizes were determined due to the number of patients who visited our clinics. from 2014 to 2019. No sample-size caluculation was performed. The differences between the groups were evaluated by Student t test, Mann-Whiney U test or two-way ANOVA using GraphPad Prism 7.0 Software. A value of P < 0.05 was considered to be statistically significant.
Data exclusions	No data were excluded from the study.
Replication	All experimental findings in our experiments using mice were replicated. The analyses on human samples cannot be reproduced.
Randomization	No prospective clinical study was performed in our study. All mice at same ages from the same faculty were allocated into experimental groups.
Blinding	Assessments were conducted by an assessor blind to group allocation.

Reporting for specific materials, systems and methods

We require information from authors about some types of materials, experimental systems and methods used in many studies. Here, indicate whether each material, system or method listed is relevant to your study. If you are not sure if a list item applies to your research, read the appropriate section before selecting a response.

Materials & experimental systems

n/a	Involved in the study	
☐	☒	Antibodies
☒	☐	Eukaryotic cell lines
☒	☐	Palaeontology and archaeology
☐	☒	Animals and other organisms
☐	☒	Human research participants
☐	☒	Clinical data
☒	☐	Dual use research of concern

Methods

n/a	Involved in the study	
☒	☐	ChIP-seq
☐	☒	Flow cytometry
☒	☐	MRI-based neuroimaging

Antibodies

Antibodies used	Functional antibodies used in vivo studies: Anti-murine interleukin-6 receptor blocking antibody (clone MR16-1) provided by Chugai Pharmaceuticals; anti-murine interleukin-17A neutralizing antibody (clone 17F3, Catalog# BE0173) and anti-murine PD-1 antibody (clone RMP1-14, Catalog# BE0146) provided by Bio X Cell<br>Staining antibodies used in Histological analysis and Flow cytometry analysis: Anti-human CD8 and anti-human CD4 monoclonal antibodies (clone C8/144B and 4B12, Catalog # 413211 and 413961) provided by Nichirei Biosciences; anti-murine CD3 (clone SP7, Catalog# ab16669), goat anti-rabbit IgG polyclonal antibody (Catalog# ab150077), and goat anti-rat IgG polyclonal antibody (Catalog# ab150158) provided by Abcam; anti-murine CD8a antibody (clone 4SM15, Catalog# 50-112-9056), anti-B220 antibody (clone RA3-6B2, Catalog#12-0452-83), and anti-Gzm B antibody (clone NGZB, Catalog#11-8898-82) provided by eBioscience; anti-murine FcγIII/II receptor antibody (clone 2.4G2, Catalog# 553140) and anti–IFN-γ (clone XMG1.2, Catalog#554413) provided by BD; anti-murine CD45 antibody (clone 30-F11, Catalog#562420), anti-murine CD4 antibody (clone GK1.5, Catalog#100443), anti-murine CD8a antibody (clone 53–6.7, Catalog#100766), anti-murine CD3e (clone 145-2C11, Catalog#100348), and anti-PD-1 antibody (clone 29F.1A12, Catalog#135210) provided by BioLegend
Validation	MR16-1, Okazaki M, et al. Immunol Lett. 2002; 84(3): 231-240; We used other antibodies by referring to manufacturer's website.

Animals and other organisms

Policy information about studies involving animals; ARRIVE guidelines recommended for reporting animal research

Laboratory animals	C57BL/6 background male mice, 8 to 12-weeks-old
Wild animals	The study did not involve wild animals.
Field-collected samples	The study did not involve samples collected from the field.
Ethics oversight	All patients provided written, informed consent in compliance with the approval by the Institutional Ethics Committee at the University of Tsukuba Hospital (number: H28-045 and H30-256). All animal experiments were approved by the Animal Experiment Committee of the University of Tsukuba (Permit Number: 17–137), and performed in accordance with the Guide for the Care and Use of Laboratory Animals of the University of Tsukuba.

Note that full information on the approval of the study protocol must also be provided in the manuscript.

Human research participants

Policy information about studies involving human research participants

Population characteristics	Skin samples were obtained from melanoma (n = 3), renal cell carcinoma (n = 2), gastric cancer (n = 1) and lung cancer (n = 1) patients with anti-PD-1 antibody-induced psoriasis-like dermatitis (n = 7), and idiopathic psoriasis patients (n = 6), who visited Tsukuba University Hospital (Japan) and Mito Saiseikai General Hospital (Japan) from 2014 to 2018. Serum samples were collected post-treatment from melanoma (n = 25), renal cell carcinoma (n = 1) and lung cancer (n = 1) patients treated with anti-PD-1 antibody at Tsukuba University Hospital (Japan) from 2014 to 2019 (n = 27), including eight patients who developed psoriasis-like dermatitis after the treatment.
Recruitment	A single- (or two-) center retrospective observation study
Ethics oversight	All patients provided written, informed consent in compliance with the approval by the Institutional Ethics Committee at the University of Tsukuba Hospital (number: H28-045 and H30-256).

Note that full information on the approval of the study protocol must also be provided in the manuscript.

Clinical data

Policy information about clinical studies

All manuscripts should comply with the ICMJEguidelines for publication of clinical research and a completedCONSORT checklist must be included with all submissions.

Clinical trial registration	N/A
Study protocol	N/A
Data collection	N/A
Outcomes	N/A

Flow Cytometry

Plots

Confirm that:

- [ ] The axis labels state the marker and fluorochrome used (e.g. CD4-FITC).
- [x] The axis scales are clearly visible. Include numbers along axes only for bottom left plot of group (a 'group' is an analysis of identical markers).
- [ ] All plots are contour plots with outliers or pseudocolor plots.
- [x] A numerical value for number of cells or percentage (with statistics) is provided.

Methodology

Sample preparation	Draining lymph nodes were harvested from mice, and single-cell suspensions were prepared by mashing.
Instrument	Gallios 10 Colors, 3 Lasers (B5-R1-V2 Configuration, Beckman-Coulter)
Software	Kaluza (Beckman-Coulter) was used for collection, and FlowJo software (v7.6.5, BD) was used for analysis.
Cell population abundance	For analysis of the cell surface markers, more than two millions viable cells were used. For intracellular staining analysis, more than one million viable cells were used.
Gating strategy	We gated to measure viable cells using FSC and SSc, and gated FSC-W and FSC-A for identification of single cells. Then, we finally determined viable cell population using the Zombie fixable viability kit (BioLegend). Fluorescence-minus-one controls were used as negative controls to determine where boundaries between positive and negative.

- [ ] Tick this box to confirm that a figure exemplifying the gating strategy is provided in the Supplementary Information.
